# 

**Published:** 1852-07

**Authors:** 


### CONTENTS.

### JOURNAL.

Page.

ARTICLE I.—Treatment of Dental Caries, complicated with Disorders of the Pulp and Peridental Membrane. No. V. By Robert Arthur, D. D. S., M. D. . . . . . . 505

ARTICLE II.—Opening Address, delivered before the American Society of Dental Surgeons at the Annual Meeting held in Philadelphia, August, 1851. By J. H. Foster, M. D. . 532

ARTICLE III.—The Relation of Tic Douloureux to disorders of the Teeth. By Toogood Downing, M. D. . . . . . 556

ARTICLE IV.—On Amalgam Stoppings. By W. R. Bridgman, . 561

ARTICLE V.—Dental Inventions and Pretensions, . . . 567

ARTICLE VI.—Dental Exhibition at the World's Fair, . . 571

ARTICLE VII.—Tartar on Teeth, . . . . . . . 577

ARTICLE VIII.—On Materials the best adapted for Filling the Teeth. By Wm. Robertson, Esq. . . . . . . . 580

ARTICLE IX.—On a New Soldering Apparatus. By Spence Bate, 587

ARTICLE X.—Gratuitous Services Injurious when given without due discrimination. . . . . . . . . . 590

ARTICLE XI.—Patents in any of the Different Branches of Medicine. By W. R. Handy, M. D., . . . . . . . 594

### SELECTED ARTICLES.

ARTICLE XII.—Teeth—Comparative Anatomy, . . . 600

ARTICLE XIII.—Acute Periostitis. By J. H.M'Quillen, M.D., Dentist, 646

ARTICLE XIV.—Remarks on Atmospheric Plates. By T. L. Buckinghan, M. D., Dentist, . . . . . , . . 652

ARTICLE XV.—Illustrations of the successful treatment of Cleft Palate by the Division of the Levator-Palati, Palato-Pharyngeus, and sometimes the Palato-Glossus Muscles. By John Avery, Esq., F. R. S., Surgeon to Charing-Cross Hospital, . 658

### EDITORIAL DEPARTMENT.

Dr. Gardette and his Proposition Again, . . . . . . 669

Ohio College of Dental Surgery, . . . . . . . 671

Medical Department of the Pennsylvania College, . . . 671

Philadelphia College of Dental Surgery, . . . . . 671

Baltimore College of Dental Surgery, . . . . . . . 672

Dr. W. H. Dwinelle, . . . . . . . 672

Bond's Dental Medicine, . . . . . . . . . . 672

